# Ghana: Accelerating neglected tropical disease control in a setting of economic development

**DOI:** 10.1371/journal.pntd.0007005

**Published:** 2019-01-17

**Authors:** Peter J. Hotez, Nana-Kwadwo Biritwum, Alan Fenwick, David H. Molyneux, Jeffrey D. Sachs

**Affiliations:** 1 Texas Children’s Hospital Center for Vaccine Development, Departments of Pediatrics and Molecular Virology and Microbiology, National School of Tropical Medicine, Baylor College of Medicine, Houston, Texas, United States of America; 2 Center for Medical Ethics and Health Policy, Baylor College of Medicine, Houston, Texas, United States of America; 3 Department of Biology, Baylor University, Waco, Texas, United States of America; 4 James A Baker III Institute of Public Policy, Rice University, Houston, Texas, United States of America; 5 Scowcroft Institute of International Affairs, Bush School of Government and Public Policy, Texas A&M University, College Station, Texas, United States of America; 6 Bill & Melinda Gates Foundation, Seattle, Washington, United States of America; 7 Ghana Health Service, Accra, Ghana; 8 Department of Infectious Disease Epidemiology, Imperial College London, United Kingdom; 9 Liverpool School of Tropical Medicine, Liverpool, United Kingdom; 10 Center for Sustainable Development, Earth Institute, Columbia University, New York, New York, United States of America; University of Nottingham, UNITED KINGDOM

## Introduction

Ghana is exhibiting impressive economic gains that may compare with the growth rates expected in India or China. With economic development, there is an expectation that the prevalence and disease burden of the neglected tropical diseases (NTDs) and other poverty-related neglected diseases will decline. Indeed, guinea worm, human African trypanosomiasis, and trachoma recently have been eliminated in Ghana, and there have been steep declines in the prevalence of onchocerciasis and lymphatic filariasis (as well as oesophogostomiasis and yaws), with the prospect of eliminating these diseases as well in the not-too-distant future. In contrast, progress toward disease prevalence reductions for schistosomiasis, hookworm, and other soil-transmitted helminth infections, as well as other NTDs, including cysticercosis, cystic echinococcosis, scabies, Buruli ulcer, and leprosy, have been more modest. Snake bite envenoming, an important regional noninfectious NTD, also requires a different strategic approach. Arbovirus infections are emerging and thus remain a significant and under-recognized public health threat. For some of these NTDs, new technologies, including vaccines, will be required. Health-system strengthening with mobile health-activities are expected to continue furthering NTD disease reductions, with the hope that Ghana could become the first highly populated Sub-Saharan African nation to achieve its NTD elimination targets.

## Overview

**Fig 1:**
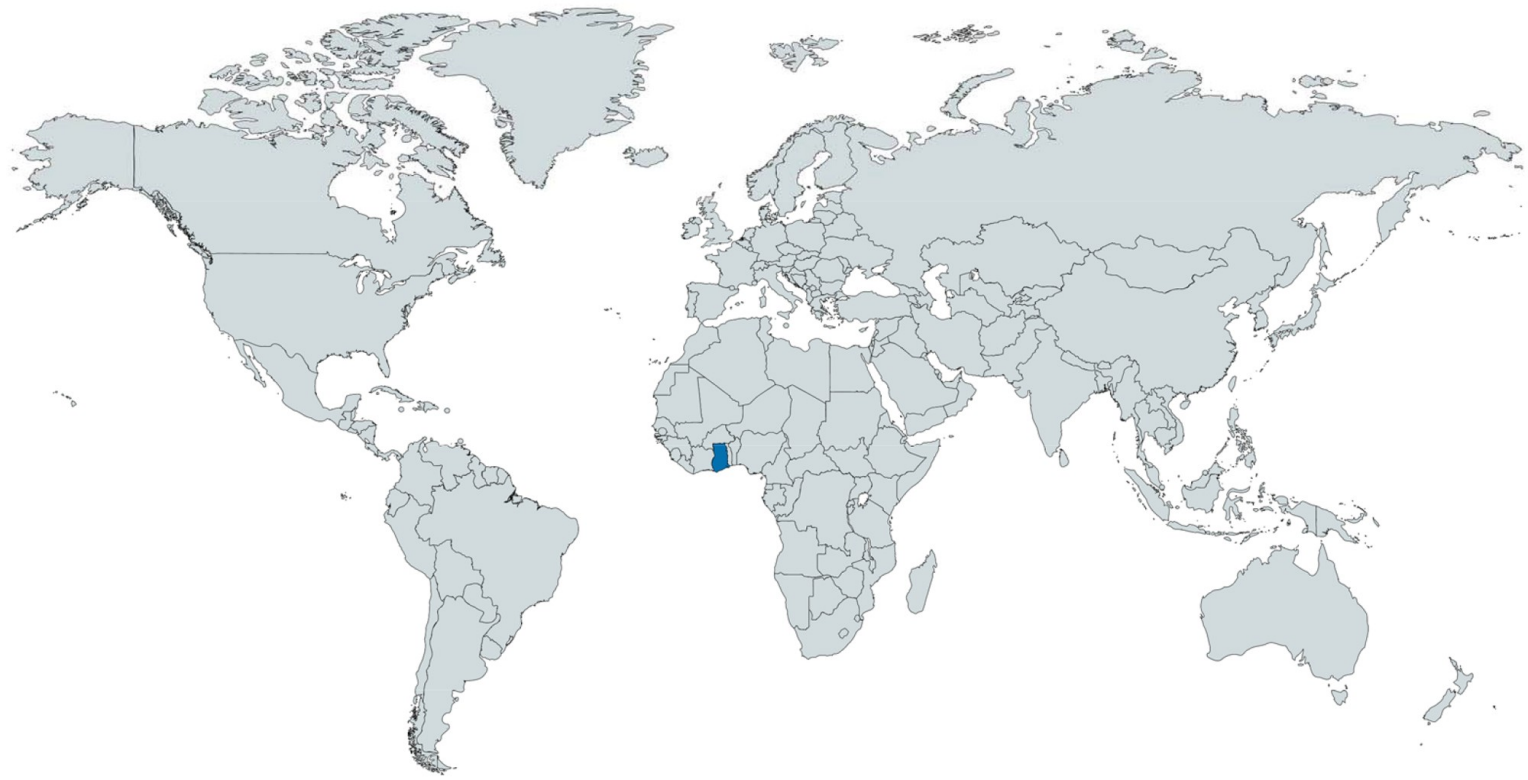
The nation of the Republic of Ghana. Original figure made with mapchart.net.

The Republic of Ghana occupies a land mass of 238,500 square kilometers with a rising population now approaching 30 million people (Fig 1) [1]. Ghana is showing signs of impressive economic growth. It is estimated that approximately one quarter (24.2%) of Ghanaians lived below the national poverty line as of 2013, down from 56.5% in 1992 with reductions in urban poverty achieving the greatest gains [2]. Indeed, recent accounts suggest that Ghana currently boasts a relatively high annual growth rate [3], expected to be in the range of 5% to 9% per annum in coming years, roughly comparable to rates expected for China or India. For example, the World Bank and the African Development Bank each estimate that Ghana’s economy will accelerate to between 8% and 9% in 2018, with some decrease of the growth rate in subsequent years [3–5].

One of the most potent effects of rapid economic development and improvement is the reduction in parasitic and related NTDs [6]. The exact mechanisms that inversely link rising economies to NTDs remain to be fully understood, but the relationships represent important late 20th century and early 21st century narratives [7]. For example, with the rapid economic improvements in Eastern China over the last two decades, there have been steep declines in helminth infections, whereas these diseases largely remain highly prevalent in China’s impoverished southwestern regions [7,8]. Aggressive economic improvements following World War II in Japan and South Korea have also been linked to historic decreases in helminthiases and other NTDs [7].

For these reasons, it is of interest to see if Ghana’s recent economic growth is translating into public health gains through reductions in NTDs. Shown in Table 1 are results from the Global Burden of Disease (GBD) Study 2017 that compare prevalence (parasitic and bacterial NTDs) or incidence (dengue) rates of some of the major NTDs, both at the launch of the Millennium Development Goals (MDGs) and then in the year 2017 [9].

**Table 1:** Comparison of prevalence and incidence of selected NTDs in Ghana in the years 2000 and 2017 [9].

Parasitic and bacterial NTDs	Estimated prevalence in 2000	Estimated prevalence in 2017	Percentage change 2000–2017
Hookworm	2,741,253	2,993,431	+09%
Schistosomiasis	1,416,722	968,264	-32%
Ascariasis	853,804	897,167	+05%
Lymphatic Filariasis	744,181	59,929	-92%
Trichuriasis	351,952	491,130	+40%
Onchocerciasis	91,456	5,987	-93%
Guinea worm	3,345	0	-100%
Leprosy	1,078	800	-26%
Viral NTDs	Estimated incidence in 2000	Estimated incidence in 2017	
Dengue	70,526	308,811	+338%

**Abbreviation:** NTD, neglected tropical disease.

## NTDs targeted for elimination

Among the most significant NTD progress in Ghana since the launch of the MDGs have been the certified eliminations of guinea worm transmission and trachoma and the near elimination of human African trypanosomiasis, as well as dramatic reductions in the prevalence and intensity of onchocerciasis and lymphatic filariasis (LF). For each of these NTDs, however, it is difficult to attribute these public health successes directly to economic development instead of concerted and targeted vertical intervention programs assisted by donor support.

Ghana’s guinea worm eradication program was launched in 1988 as a partnership with the Carter Center, UNICEF, and WHO [10]. Although almost 200,000 cases were initially reported a year after the launch, through aggressive programs of providing access to safe water, chemical treatments against copepods, health education, case containment, establishment of a reward system, and improved surveillance and reporting, the transmission of guinea worm disease was halted in 2010, and the absence of transmission was certified by WHO in 2015 [10]. The progress leading to elimination was achieved despite a temporary setback due to ethnic conflicts in northern Ghana and an “epidemic” event in which water supplies were disrupted in a major city and contaminated drinking water was supplied to the population resulting in a surge of cases [10].

Similarly, the Carter Center’s Trachoma Control Program initiated work with the Ghana Health Service to train community health workers and launch programs of health education and sanitation, while implementing the SAFE (Surgery, Antibiotic mass treatment with azithromycin, Face washing and Environmental improvement) strategy, such that by 2008 Ghana became the first nation in Sub-Saharan Africa to meet critical elimination targets, with every community with trachoma receiving SAFE interventions for three years or more [11, 12]. However, follow-up surveys indicated that trachoma, although greatly reduced, was still not entirely eliminated, and efforts were required to pivot away from mass treatments in order to focus on post-treatment surveillance and on case detection and treatment to eliminate the backlog of trichiasis surgery [11, 12]. In subsequent years, the Ghana NTD Program in collaboration with the END in Africa project of FHI360, the International Trachoma Initiative of the Task Force for Global Health, and Sightsavers International continued this approach, such that in 2015 efforts were redoubled to push for true elimination [11–13]. From these activities, it appears that trachoma has been eliminated, with Ghana becoming the first nation in the WHO’s African region to reach this goal [14]. In addition, annual azithromycin treatments appear to have some impact on reducing the prevalence of yaws in Ghana [15].

For both onchocerciasis and LF, significant reductions in the parasitological parameters of prevalence (greater than 90% reduction) and intensity and entomological measures of transmission have been achieved initially for onchocerciasis via larviciding and later through mass drug administration (MDA) with ivermectin or ivermectin together with albendazole for lymphatic filariasis. In Ghana, the Onchocerciasis Control Program (OCP) began in 1974 through vector control activities before switching to MDA in 1998 (with the exception of the Asubende focus in which vector control continued under the OCP Special Intervention Zones) [16]. Following a mapping exercise that identified remaining endemic areas, starting in 2009, 85 of the 216 districts in Ghana began to receive mass treatments. In 2006, the NTD Program of the United States Agency for International Development (USAID) expanded support for onchocerciasis and LF control through an integrated program of MDA, which also includes the soil-transmitted helminthiases and schistosomiasis [17]. The program was led by the Ghana Health Service, which was further supported in 2010 by END in Africa [16]. In some areas, ivermectin is delivered twice a year in a biannual treatment strategy [18]. A significant concern, however, is that as adult *Onchocerca volvulus* worms can live for more than a decade, so ongoing MDA will be required for some years to come. In addition, a study in Ghana in which ivermectin had been used for many years showed that there was an increase in the rate of repopulation of the skin, suggesting that there was reduced efficacy of the drug [19]. However, according to the GBD 2017, more than a 90% reduction has been achieved. A recent impact assessment indicates that MDA has had significant effect such that control efforts for onchocerciasis can transition toward an elimination strategy. In so doing, areas of the countries that became “hypo-endemic” as a result of MDA are being retargeted. The GBD studies may not fully consider those populations who remain afflicted by irreversible blindness and disfiguring skin disease—a situation, which is paralleled in cutaneous leishmaniasis; only active infections are considered by the GBD metrics, excluding those with residual “inactive” disease [20].

LF is simultaneously targeted through ivermectin and albendazole MDA, resulting in significant prevalence reductions such that mass treatment is no longer needed in many health districts. LF control and elimination efforts first began during the late 1990s, following a World Health Assembly resolution [21–24], and accelerated after an extensive mapping exercise conducted jointly by the Ghana Health Service and the Liverpool School of Tropical Medicine in 2001 [23] through support from the United Kingdom Department for International Development. Overall, it is estimated that more than 13 million people in Ghana no longer require MDA. However, a number of important LF “hotspots” remain and will continue to require treatment [24, 25]. Some investigators consider that Ghana is on the verge of LF elimination in urban areas of Ghana and elsewhere in West Africa, so that MDA could be replaced with case surveillance and treatment [26]. It has also been noted that the use of albendazole and ivermectin in the LF and onchocerciasis programs has been attributed to the decline—and possibly elimination—of a soil-transmitted helminth infection, oesophagostomiasis, which is seemingly unique to northern Ghana and Togo [27–29].

In summary, the combined effects of economic development and MDA are leading to rapid decreases in the prevalence and transmission of onchocerciasis, LF, oesophagostomiasis, and yaws, whereas guinea worm infection and trachoma have already been eliminated in Ghana. No cases of human African trypanosomiasis (a historic scourge) have been reported over recent years, suggesting it has also been eliminated [30], possibly due to the expanding rural population and increased pressure on available land resource resulting in the drastic decline in *Glossina palpalis* tsetse habitats. Overall, if these trends and activities are sustained, there is optimism that Ghana could lead countries of Sub-Saharan African in terms of NTD elimination efforts with success achieved sometime in the 2020s.

## NTDs that remain widespread

In contrast to the public health successes highlighted above, for other high prevalence NTDs, the gains appear to be more modest. According to the GBD 2017, schistosomiasis is the second most prevalent human helminth infection (following hookworm infection) in Ghana, affecting almost one million people [9]. *Schistosoma haematobium*, the cause of urogenital schistosomiasis is the predominant human schistosome, but *Schistosoma mansoni*, the cause of intestinal schistosomiasis, is also prevalent [31, 32]. The prevalence of schistosomiasis increased sharply following the creation of Lake Volta after the construction of the Akosombo hydroelectric dam during the 1960s [33]. Before 2007, schistosomiasis control relied primarily on snail control, especially in the basin of the Volta River [31]. In 2008, USAID began supporting programs of MDA, which included mass treatments with praziquantel [34]. Disease mapping and MDA programs have accelerated in the last five years for schistosomiasis (and also the major soil-transmitted helminth infections) [31–35]. With respect to the former, impact assessments conducted in 2016 show overall reductions in endemicity, although population increases in many of the endemic areas have maintained or even increased estimates of the number of people considered at risk for schistosomiasis. According to the GBD 2017, the overall prevalence of schistosomiasis in Ghana has decreased by approximately one-third since 2000 [9]. Pending significant declines in schistosomiasis prevalence, it is possible that a control strategy might be switched to case detection using sensitive diagnostics and then treatment. Two additional platyhelminth infections, cysticercosis and cystic echinococcosis, also remain endemic.

All three major soil-transmitted helminthiases—hookworm infection, ascariasis, and trichuriasis—are also widespread in Ghana, especially among pediatric populations. Whereas MDA appears to have been effective at reducing the transmission of LF and onchocerciasis in Ghana, the GBD 2017 suggests that this approach has not been as successful in diminishing the prevalence of soil-transmitted helminth infections. However, given the extensive distribution of albendazole and ivermectin for LF and onchocerciasis programs over the last 20 years, it would be expected that indeed both the prevalence and intensity of soil-transmitted helminth infections may also be starting to decline. Of the three, hookworm infection is the predominant soil-transmitted helminth infection, both according to GBD 2016 and field based investigative studies [36–38]. In Ghana, hookworm infection has been linked to serious malnutrition among both children and adults, and it has been noted that endemic hookworm is often refractory to single dose albendazole MDA [36–38]. Indeed, reduced albendazole susceptibility among hookworm isolates in Ghana following treatment has suggested the possibility of emerging albendazole drug resistance [37]. However, underlying nutritional status may also be a factor in the varying levels of albendazole drug efficacies [38]. Also related to hookworm and malnutrition is the high incidence and prevalence of hookworm and malaria coinfections, leading to severe anemia [36–38]. Indeed, comorbidities from malaria and a wide range of childhood infections are common [39].

Other NTDs that remain endemic include Buruli ulcer [40, 41]. During the early 2000s, almost 6,000 patients were identified [40], although initiatives are underway to promote early case detection and treatment through community-based surveillance volunteers [42]. Recent application of geographic information systems has identified Buruli ulcer “hotspots” along specific riverine areas that might be targeted for enhanced surveillance and treatment [43]. Ghana has been at the forefront of the development of a new WHO-approved treatment strategy for buruli ulcer based on oral antibiotic therapy [44] as well as studies on the social and mental health burden of this condition [45]. Leprosy also remains prevalent, although the numbers of cases have dramatically declined since the introduction of multidrug therapy [46]. Additional and important NTDs include scabies and snake-bite envenomation.

Finally, among the arbovirus infections, the incidence of dengue in Ghana and elsewhere in West Africa is believed to have risen precipitously. Dengue risk appears to be the highest in coastal areas [47]. One of the major issues affecting dengue in Ghana is the lack of disease surveillance efforts, especially in febrile patients who are presumed to be infected with malaria [47–49]. According to WHO, Ghana is one of 14 countries considered at high risk of yellow fever outbreaks, although it is not currently one of the four West African nations considered as a “complex emergency situation” [50].

## Future directions and call to action

The extent to which Ghana’s economic gains have translated or are directly related to reductions in NTD prevalence and incidence remains to be determined. Ghana has successfully eliminated guinea worm and trachoma (certified by WHO) and is on a path to eliminate some additional high profile NTDs such as yaws, onchocerciasis, and LF. The dramatic reduction in urban poverty suggests that any urban foci of these diseases would be the first to disappear, possibly even without MDA. However, across the rural areas of Ghana, schistosomiasis and soil-transmitted helminthiases, especially hookworm infection, remains widespread, and dengue appears to be on the rise.

Ghana is on a good trajectory in terms of providing access to NTD essential medicines and healthcare, with the recent adoption of mobile health (mhealth) practices and other innovations that include several sectors of the economy. A road map is in place to scale-up successful models that demonstrate adequate value. Going paperless is an important advance in the health systems, with mhealth taking the lead in scalable technologies to support healthcare delivery despite challenges associated with security, open sourcing, and appropriate software design. Mhealth is currently being used to provide feedback on blood stocks and verbal autopsies as well as support the District Health Information Systems Version 2. Community health workers (CHWs) are being deployed nationally to provide various primary health services and increasingly are utilizing mhealth systems. In addition, over the last two decades, there has been an impressive increase in human capacity dedicated to research on NTDs supported from both within Ghana and via external contributions thereby strengthening individual and institutional research programs on many of the NTDs together with the establishment of the Secretariat of African Research Network for NTDs.

Based on past trajectories in several East Asian countries, in which MDA went hand-in-hand with aggressive economic development, we should start to see steep declines in all of Ghana’s NTDs, leading to elimination of some currently high-profile diseases. For schistosomiasis and soil-transmitted helminthiases, there is also some early progress, although an expanding population in at-risk areas makes public health gains more problematic to assess. The concerns about albendazole resistance and reduced ivermectin efficacy will also need to be addressed, with consideration of developing improved biotechnologies for some of the NTDs [51]. Moreover, we have seen how dengue and yellow fever can persist even in middle-income countries, so that for these diseases as well new technologies will need to be developed and implemented. With an appropriate focus on mhealth and community-based health services, including the national deployment of CHWs supported by mhealth applications, Ghana could become the first Sub-Saharan African nation with a large population to achieve widespread success in NTD control and elimination. With targeted and scaled-up efforts, Ghana has the potential to eliminate the major NTDs over the next decade.
